# Impact of Strict Isolation Precautions on Families with a Language Other than English

**DOI:** 10.1089/heq.2024.0117

**Published:** 2025-01-29

**Authors:** Polina Frolova Gregory, Sanyukta Desai, Corrie E. McDaniel, Austin DeChalus, Emily Bowen, Michael Dinh, Jessica Gagen, Dwight Barry, Abena Knight, Matthew Test, Arti D. Desai, Mersine A. Bryan

**Affiliations:** ^1^ Department of Pediatrics, University of Washington School of Medicine, Seattle, Washington, USA.; ^2^ Seattle Children’s Research Institute, Seattle, Washington, USA.; ^3^ The University of Texas at Austin Dell Medical School, Austin, Texas, USA.; ^4^ Children’s Hospital of Philadelphia, Philadelphia, Pennsylvania, USA.; ^5^ University of Washington School of Medicine, Seattle, Washington, USA.; ^6^ Department of Clinical Analytics, Seattle Children’s Hospital, Seattle, Washington, USA.

**Keywords:** interpreter use, isolation precautions, LOE

## Abstract

**Introduction:**

Children with a language for care other than English (LOE) are at risk for inequitable care. We examined the association of isolation precautions in the care of hospitalized children with LOE through the frequency of professional interpreter use and timing of in-person consultation.

**Methods:**

Retrospective cohort study of children in a strict isolation unit (SIU) between 2/2020 and 12/2021. Negative binomial regression was used to assess both differences in interpretation rates between SIU and non-SIU, and within 72-h/in-person consultation rates within the SIU between English-speaking and LOE encounters.

**Results:**

We identified 487 encounters in the SIU; 126 (26%) involved patients with LOE. The median interpretations per day were 4.5 (Interquartile Range [IQR]: 2.0–6.7). Among patients with LOE, there was an observed difference in median interpretations per day in the SIU (3.9, IQR: 1.7–6.4) versus encounters in non-SIU acute care units (5.0, IQR: 1.2–8.2). However, this difference was not statistically significant (Incidence Rate Ratio [IRR]: 0.89; 95% confidence interval [CI]: 0.70–1.13). Sub-specialty consultations were requested for 410 (84%) encounters within the SIU; 282 (69%) were completed in person within 72 h. A small difference between the percentage of completed consultations for encounters with LOE (*n* = 61, 64%) and English-speaking patients’ encounters (*n* = 221, 70%) was noted, which again was not statistically significant (IRR: 0.93; 95% CI: 0.71–1.21).

**Conclusion:**

Despite the increased barriers of strict isolation, we exceeded institutional standards for interpretations per day and had similar rates of interpretation for encounters with LOE admitted to medical units regardless of isolation status.

## Introduction

Children in families with caregivers who speak a primary language for medical care other than English (LOE) are at risk of experiencing medical errors, serious adverse medical events, and increased hospital length of stay (LOS).^1^ Inadequate communication by health care providers during hospitalization and the underuse of professional interpretation services are important contributors to inequitable care.^2^

Insufficient communication during health care encounters with caregivers who speak a LOE has been described in varied health care settings. These settings include interactions in the emergency department, intensive care units, acute medical care units, and outpatient clinics.^3–8^ During these health care encounters, children of parents with LOE have been shown to experience decreased health care access,^9^ longer duration for visits within the emergency department setting, and higher resource utilization for diagnostic testing.^4^ Prior studies reported that only 73% of families with LOE received any interpretation services upon admission to a pediatric intensive care unit^6^ and only half received professional interpretation services during a given ED or outpatient encounter.^10,11^ Despite increased awareness of inequitable communication across these encounters, there remains a limited understanding of how professional interpretation services are utilized in a strict isolation setting, where access to professional interpreters may be even more limited. When patients with highly communicable diseases are hospitalized, they are often placed under strict isolation precautions that include the use of single negative pressure rooms where clinicians wear gowns, gloves, and N-95 masks or controlled air-purifying respirators (CAPR).^12^ The structural limitations to strict isolation, including caregiver ability to participate in family-centered rounds, potential shortages in personal protective equipment (PPE), and unreliable access to professional interpretation, may further increase inequities in communication and perpetuate disparities in the care of children with a LOE.^5^

Our objective was to assess two measures for children with LOE that may be impacted by strict isolation precautions: (1) the frequency of interpreter use and (2) timing to completion of in-person consultation. Both measures were compared to our institutional standards for these services. We hypothesized that (1) the number of interpretations per day for LOE patients in the strict isolation unit (SIU) would be lower than rates for LOE patients admitted to non-SIU medical units and (2) the proportion of in-person consultation within 72 h would be lower for LOE patients compared to English-speaking patients among those in the SIU.

## Methods

### Study design

We conducted a retrospective cohort study using electronic health record (EHR) data and medical record review of children hospitalized at a freestanding children’s hospital between 2/2020 and 12/2021. Our institution provides medical care to children between the ages of 0 and 21 years. Institutional Review Board approval was obtained.

### Setting and study population

In March 2020, our hospital established an SIU for patients with confirmed or suspected SARS-CoV-2 to reduce the number of individuals entering a patient room while maintaining patient safety. All SIU rooms were negative pressure rooms; health care personnel were required to follow guided PPE donning and doffing procedures for eye protection, CAPR or N-95 masks, gowns, and gloves. Initially, the nurse-to-patient ratio was set to 1 nurse:2 patients and increased to our institutional standard ratio of 1:3 after 6 months. Clinical teams initially included 1 supervising physician and 1 resident physician, and within 2 months expanded to a full team composed of 5–6 individuals. Clinicians entered rooms to perform medically necessary exams but were encouraged to hold extended conversations with patients and caregivers by phone or telehealth when possible. In-person interpreters were not permitted to enter SIU rooms; all professional interpretation services were converted to iPad telephone/video interpretation. Phone and video interpretation at our hospital is available 24 h/day. During the study period, 67 institutional providers were certified for medical interpretation in an LOE, with 2 providers certified for interpretation in two LOE. Designated iPad stations were provided in all SIU rooms for families with LOE.

We included all children ≤18 years of age. Encounters in the SIU were identified using room numbers within the defined physical space.

### Data collection

Two investigators reviewed potential encounters to ensure they met inclusion criteria. We obtained demographic variables, LOS, level of medical complexity using the Pediatric Medical Complexity Algorithm (PMCA),^13^ and number of interpretations per day from hospital administrative data. Each interpretation encounter is linked to a patient’s medical record number and date. Interpretation encounters included communication initiated by any clinical team member (e.g., clinicians, nurses, dietitians, therapists, social workers). Interpretation charges from vendor invoices were matched to patients using medical record numbers, date, and time. We conducted a medical record review to identify the timing of an in-person consultation, defined as the time from when the consult order was signed in the EHR compared to the time when the clinician completed a consult note. Data were abstracted into a Research Electronic Data Capture (REDCap) database.^14^

### Outcome measures

For the first measure, we calculated the number of interpretations per day for patients with LOE in the SIU and among patients admitted to non-SIU medical units contemporaneously using hospital administrative data. For the second measure, we calculated the proportion of patients who had an in-person consult within 72 h for patients in the SIU stratified by language for care. Our institutional standards for these two measures are: (1) ≥ 2 interpreter uses per day (telephone, video, or in person) for patients with LOE based on prior institutional quality improvement work^15^ and (2) in-person consultation completed within 72 h based on our intra-institutional policy standard of 48 h^16^ while allowing an additional 24 h to account for weekend consultation requests.

### Statistical analysis

Descriptive statistics were used to characterize the median (IQR) number of interpretations per patient per day and the proportion (n) of encounters that required a subspecialty consult. We calculated the consultation and interpretation rate per day as the total number of consults or interpretations that took place during a patient’s hospitalization divided by the length of stay in fractional days. Finally, we used negative binomial regression models to examine differences in interpretation counts for encounters with LOE in strict isolation versus standard precautions, and to compare counts of encounters with ≥1 in-person consult completed within 72 h for LOE versus English-speaking patients among patients in the SIU. Both models used length of stay as an exposure variable, to account for different LOS times across encounters. The consult model adjusted for PMCA category. Model fit comparisons and assessments showed that mixed-effects models were not required to account for the small number of patients (11) with multiple encounters. All analyses were performed using R version 4.4.

## Results

We identified 458 patients with 487 encounters in the SIU (Table 1). A total of 11 patients in our cohort had multiple encounters.

**Table 1.** Demographic and Clinical Characteristics of Patients Admitted to the SIU

	English	LOE**	
Characteristic*	SIU, *n* = 361	Non-SIU, *n* = 135	SIU, *n* = 126
Age (years)	10.0 (3.0, 15.0)	5.0 (1.0, 12.0)	9.5 (2.0, 15.0)
Legal Sex			
Female	180 (50)	64 (47)	62 (49)
Male	180 (50)	71 (53)	64 (51)
X (non-binary)	1 (0.5)		
Race			
2 or more races	16 (5)	1 (0.5)	6 (5)
American Indian	1 (1)	1 (0.5)	0
Asian	21 (6)	15 (11)	14 (11)
Black or African American	40 (11)	21 (15)	12 (10)
Other	60 (17)	74 (55)	70 (55)
Pacific Islander	2 (1)		
Patient Refused	21 (6)	4 (3)	6 (5)
Unknown	1 (1)		
White	187 (52)	19 (14)	17 (14)
Ethnicity			
Hispanic	86 (24)	77 (57)	86 (68)
Non-Hispanic	258 (71)	54 (40)	37 (29)
Patient Refused	16 (4)	3 (2)	3 (3)
Unavailable or Unknown	1 (1)	1 (1)	0
PMCA			
Complex Chronic	172 (48)	48 (35)	62 (49)
Non-chronic	118 (32)	51 (38)	38 (30)
Non-complex Chronic	71 (20)	36 (27)	26 (21)

*Median (IQR); *n* (%).

**Languages in this study population included: Amharic, Arabic, Brazilian Portuguese, Burmese, Cambodian, Dari, Egyptian Arabic, English, Farsi; Persian, French, Karen, Mandarin, Marshallese, Mongolian, Nepali, Oromo, Portuguese, Quiche, Russian, Sign-ASL, Somali, Spanish, Swahili; Kiswahili, Tagalog, Tigrinya, Ukrainian, Vietnamese, Wolof.

ICU, intensive care unit; IQR, interquartile range; LOE, language other than English; PMCA, Pediatric Medical Complexity Algorithm; SIU, Special Isolation Unit.

There were 126 encounters in the SIU and 135 in non-SIU medical units for patients with LOE. The median interpretation per patient per day was 4.5 (Interquartile range [IQR]: 2.0–6.7). There was a slight difference in the median number of interpretations per day for non-SIU (5.0, IQR: 1.2, 8.2) versus SIU encounters (3.9, IQR: 1.7, 6.4) among patients with LOE (Table 2). A negative binomial model found an incident rate ratio (IRR) of 0.89 (95% confidence interval [CI]: 0.70, 1.13) for the SIU group compared to the non-SIU group (Table 3).

**Table 2.** Interpretations per Day for SIU and non-SIU Encounters

Characteristic*	Non-SIU, *n* = 135	SIU, *n* = 126
Interpretations per Encounter	11 (4, 21)	15 (6, 27)
Interpretations per Day	5 (1.2, 8.2)	3.9 (1.7, 6.4)
Consults per Encounter	—	1 (0, 3)
Consults per Day	—	0.2 (0.0, 0.5)
Length of Stay (hours)	63.3 (32.4, 122.5)	100.5 (47.2, 193.8)
ICU Stay		
Yes	32 (24)	40 (32)

*Median (IQR); *n* (%).SIU: Special Isolation Unit, ICU: Intensive Care Unit.

**Table 3.** Negative Binomial Regression Examining Differences in Interpretation Rates and Completed Consults

Model	Parameter	β	95% CI	IRR	95% CI	*p* value
Interpretations						
	(Intercept)	1.6	1.5, 1.8			<0.001
	SIU Status					
	Non-SIU	—	—			
	SIU	−0.12	−0.35, 0.12	0.89	0.70, 1.13	0.32
Consults						
	(Intercept)	−1.3	−1.5, −1.0			<0.001
	Language Group					
	English	—	—			
	Language other than English	−0.08	−0.35, 0.19	0.93	0.71, 1.21	0.55
	PMCA Category					
	Non-chronic	—	—			
	Non-complex Chronic	0.09	−0.25, 0.44	1.10	0.78, 1.55	0.57
	Complex Chronic	−0.03	−0.29, 0.25	0.97	0.75, 1.28	0.85

95% CI, 95% confidence interval; IRR, Incidence Rate Ratio.

Subspecialty consultations were requested for 410 encounters in the SIU; 120 (29%) encounters required ≥2 consultations. In-person consultation occurred within 72 h 69% (*n* = 282) of the time; there was a small difference between the number of completed consultation for encounters with LOE (*n* = 61, 64%) compared with English-speaking patients’ encounters (*n* = 221, 70%). There was no statistically significant difference in the number of consults between LOE encounters and English-based encounters in the SIU, adjusting for length of stay and PMCA category (IRR: 0.93; 95% CI: 0.71, 1.21; Table 3**).**

## Discussion

For patients admitted under strict isolation, encounters with a LOE exceeded the institutional benchmark for number of interpretations per day for medical communication. However, in-person subspecialty consultations for families within the SIU were completed only two-thirds of the time within 72 h of request.

In a recent report on health equity initiatives in hospital strategic plans, culturally appropriate patient care was cited as the second most common initiative.^17^ As a growing number of hospitals continue to acknowledge the importance of intentionally addressing health equity, the provision of equitable communication across all hospital units is an important step to meet this metric. Children of caregivers with LOE, similar to that of elderly adults or those with designated power of attorney, represent a vulnerable population within the health care system. Recent literature continues to highlight inequities that patients with LOE experience during highly complex medical care including during surgical procedures,^18^ delivery of palliative care at the time of end-of-life services,^19^ and at hospital discharge.^20^ Notable published examples include only 16% of patients with LOE being provided access to professional interpretation on the day of surgical informed consent,^18^ along with proceduralists who are not fluent in a non-English language often using their limited non-English language skills to obtain preoperative informed consent from patients with LOE.^21^ In another study examining the caregiver experience of elderly patients with LOE following hospital discharge, up to 40% of caregivers themselves reported having limited English proficiency and only 12% were provided access to professional interpretation at the time that discharge instructions were given.^20^

Despite the increased barriers of strict isolation, we exceeded institutional standards for interpretations per day and had similar rates of interpretation for encounters with LOE admitted to medical units regardless of isolation status. Other studies have shown that implementation of readily available 24/7 interpretation services across diverse hospital units and health care settings including inpatient medical units, oncology care units, emergency departments, and community health centers reduces *ad hoc* non-professional interpretation and increases the use of professional interpretation services.^15,22–25^ Similarly, our institution has prioritized commitment to readily available professional interpretation services, either through in-person interpreters or by phone/video interpretation since 2011,^15,26,27^ and we hypothesize that our data may reflect ease of interpretation facilitated by the availability of video and phone interpreters stationed within each room for families with LOE under strict isolation. We further acknowledge that although there is no national standard for frequency and duration of daily interpretation services,^28^ four communications per day likely leaves room to continue improving communication to provide equitable care.

Timely completion of in-person subspecialty consultations was comparable between LOE and English-speaking families and fell below our recommended institutional standard. Provider anxiety around contracting a communicable disease and PPE shortages can influence provider’s hesitancy to engage directly with patients in strict isolation.^29,30^ Proactive procurement of PPE, active transmission surveillance, and timely training of health care personnel can improve clinician and patient safety. Although our hospital has defined expectations for completion of in-person subspecialty consultation,^3^ there is no real-time tracking metric to monitor this expectation. Future research is necessary to identify ways to improve timely consultation for patients requiring strict isolation precautions and should focus beyond defined policy measures to include real-time tracking along with audit and feedback measures to ensure completed consults.

Strict isolation precautions are a necessary component in managing highly infectious pathogens and will remain an area susceptible to inequity in patient care. Similar to the recent COVID-19 pandemic, the future emergence of novel pathogens may place patients with LOE at increased risk for experiencing health care disparities.^31^ Decreased direct clinician-patient interaction and challenges in communication are key aspects of hospital-based care that have sustained negative impact by strict isolation precautions,^32^ and require innovative solutions to mitigate.

Limitations include that this is a single-center study; findings may reflect institution-specific clinical practice and standards of care. The retrospective nature limits our ability to make inferences regarding why in-person consultation did not consistently take place. Because the assessment of consultations required extensive chart abstraction, only consultations conducted within the SIU were examined. Lastly, we do not have comparison data for the frequency of the medical team’s communication with English-speaking families.

## Conclusion

The need for reliable and effective infection prevention strategies to prevent transmission of highly infective organisms can lead to challenges in maintaining high-quality standards for patients with LOE hospitalized under isolation precautions. Despite the inherent challenges to frequent communication with patients and families posed by strict isolation precautions, we were able to exceed our institutional benchmark for number of professional interpretations per day for medical communication. However, completion of in-person subspecialty consultations for families within the SIU did not meet our hospital standards.

## Abbreviations Used

95% CI: Confidence interval
CAPR: Controlled air-purifying respirators
EHR: Electronic health record
ICU: Intensive care unit
IQR: Interquartile range
IRR: Incidence rate ratio
LOE: Language other than English
LOS: Length of stay
PMCA: Pediatric medical complexity algorithm
PPE: Personal protective equipment
REDCap: Research electronic data capture
SIU: Special isolation unit
