# Supplementary material for: Impact of Strict Isolation Precautions on Families with a Language Other than English
Source: Health Equity. 2025 Jan 29;9(1):100–5. doi: 10.1089/heq.2024.0117 (PMC11848053; doi:10.1089/heq.2024.0117)
Supplement: Supplementary Data S1 [file heq.2024.0117_supplementarydatas1.docx]

Supplementary Material for Frolova Gregory et al, *Impact of Strict Isolation Precautions on Families with a Language Other than English*

Regression Comparison and Results for Interpretations Model: Poisson vs Negative Binomial

- Poisson was overdispersed, so negative binomial was chosen

**Poisson**

## Call:

## glm(formula = N_INTERPRETATIONS_ENTIRE_VISIT ~ GROUP +

## offset(log(LOS_days)),

## family = poisson, data = demog_interp)

##

## Coefficients:

## Estimate Std. Error z value Pr(>|z|)

## (Intercept) 0.79706 0.02159 36.915 < 2e-16 ***

## GROUPSIU -0.10472 0.02768 -3.783 0.000155 ***

## ---

## Signif. codes: 0 '***' 0.001 '**' 0.01 '*' 0.05 '.' 0.1 ' ' 1

##

## (Dispersion parameter for poisson family taken to be 1)

##

## Null deviance: 7003.8 on 260 degrees of freedom

## Residual deviance: 6989.6 on 259 degrees of freedom

## AIC: 8101.1

##

## Number of Fisher Scoring iterations: 6

**Negative Binomial**

## Call:

## MASS::glm.nb(formula = N_INTERPRETATIONS_ENTIRE_VISIT ~ GROUP +

## offset(log(LOS_days)), data = demog_interp, init.theta =

## 1.196994748,

## link = log)

##

## Coefficients:

## Estimate Std. Error z value Pr(>|z|)

## (Intercept) 1.64808 0.08312 19.827 <2e-16 ***

## GROUPSIU -0.11831 0.11889 -0.995 0.32

## ---

## Signif. codes: 0 '***' 0.001 '**' 0.01 '*' 0.05 '.' 0.1 ' ' 1

##

## (Dispersion parameter for Negative Binomial(1.197) family taken to be

## 1)

##

## Null deviance: 288.14 on 260 degrees of freedom

## Residual deviance: 287.18 on 259 degrees of freedom

## AIC: 2051.3

##

## Number of Fisher Scoring iterations: 1

##

##

## Theta: 1.197

## Std. Err.: 0.105

##

## 2 x log-likelihood: -2045.339

Regression Comparison and Results for Consultations Model: Poisson vs Negative Binomial

- Poisson was overdispersed, so negative binomial was chosen

**Poisson**

## Call:

## glm(formula = consults ~ LOE + PMCA_category + offset(log(LOS_days)),

## family = poisson, data = consult_df)

##

## Coefficients:

## Estimate Std. Error z value Pr(>|z|)

## (Intercept) -1.28897 0.09064 -14.222 < 2e-16 ***

## LOELanguage other than English -0.37997 0.08894 -4.272 1.94e-05 ***

## PMCA_categoryNon-complex Chronic -0.08095 0.13049 -0.620 0.535

## PMCA_categoryComplex Chronic -0.47939 0.10055 -4.768 1.86e-06 ***

## ---

## Signif. codes: 0 '***' 0.001 '**' 0.01 '*' 0.05 '.' 0.1 ' ' 1

##

## (Dispersion parameter for poisson family taken to be 1)

##

## Null deviance: 1080.4 on 486 degrees of freedom

## Residual deviance: 1027.2 on 483 degrees of freedom

## AIC: 1783.7

##

## Number of Fisher Scoring iterations: 6

**Negative Binomial**

## Call:

## MASS::glm.nb(formula = consults ~ LOE + PMCA_category + offset(log(LOS_days)),

## data = consult_df, init.theta = 1.892881948, link = log)

##

## Coefficients:

## Estimate Std. Error z value Pr(>|z|)

## (Intercept) -1.26853 0.11714 -10.830 <2e-16 ***

## LOELanguage other than English -0.07743 0.13087 -0.592 0.554

## PMCA_categoryNon-complex Chronic 0.09452 0.16832 0.562 0.574

## PMCA_categoryComplex Chronic -0.02538 0.13418 -0.189 0.850

## ---

## Signif. codes: 0 '***' 0.001 '**' 0.01 '*' 0.05 '.' 0.1 ' ' 1

##

## (Dispersion parameter for Negative Binomial(1.8929) family taken to be 1)

##

## Null deviance: 488.94 on 486 degrees of freedom

## Residual deviance: 487.95 on 483 degrees of freedom

## AIC: 1476.3

##

## Number of Fisher Scoring iterations: 1

##

##

## Theta: 1.893

## Std. Err.: 0.285

##

## 2 x log-likelihood: -1466.332
